# Lymphocyte recovery and clinical response in multiple myeloma patients receiving interferon alpha 2 beta after intensive therapy.

**DOI:** 10.1038/bjc.1996.41

**Published:** 1996-01

**Authors:** B. C. Millar, J. B. Bell, R. L. Powles

**Affiliations:** Section of Academic Haematology, McElwain Laboratories, Institute of Cancer Research, Sutton, Surrey, UK.

## Abstract

The recovery of immunoregulatory cells in the peripheral blood of patients with multiple myeloma receiving maintenance therapy with interferon alpha 2 beta (IFN-alpha 2 beta) after intensive therapy with high-dose melphalan and autologous bone marrow or peripheral blood stem cell rescue was studied. IFN-alpha 2 beta significantly inhibited the recovery of CD3+, CD4+, CD8+, CD56+/CD3- and CD16+/CD3- lymphocytes compared with numbers found in patients who had no further post-transplant treatment, but had no effect on the recovery of CD19+ cells. Among patients who did not receive IFN-alpha 2 beta, the number of CD8+, CD56+/CD3- and CD16+CD3- lymphocytes recovered to values similar to normal volunteers with increasing time after intensive therapy, however the number of CD4+ cells remained significantly below levels found in normal volunteers. Although CD16+/CD3- and CD56+/CD3- cell numbers were reduced in patients receiving IFN-alpha 2 beta, natural killer (NK) activity was not affected. The levels of soluble interleukin 2 receptor (sIL-2R) were similar in all patients and IL-2 was not detected in any patient. At the time of writing, of the total of 69 patients, seven have relapsed, of whom three were receiving IFN-alpha 2 beta, however there was no correlation between the absolute numbers of any lymphocyte subset with imminent relapse. The data suggest that the recovery of a specific lymphocyte subset(s) in peripheral blood is unlikely to be associated with the maintenance of response after intensive therapy.


British Journal of Cancer (1996) 73, 236-240
© 1996 Stockton Press All rights reserved 0007-0920/96 $12.00

### Lymphocyte recovery and clinical response in multiple myeloma patients receiving interferon $\alpha_{2\beta}$ after intensive therapy

BC Millar, JBG Bell and RL Powles

*Section of Academic Haematology, The McElwain Laboratories, Institute of Cancer Research and the Royal Marsden NHS Trust, Sutton, Surrey, UK.*

**Summary** The recovery of immunoregulatory cells in the peripheral blood of patients with multiple myeloma receiving maintenance therapy with interferon $\alpha_{2\beta}$ (IFN-$\alpha_{2\beta}$) after intensive therapy with high-dose melphalan and autologous bone marrow or peripheral blood stem cell rescue was studied. IFN-$\alpha_{2\beta}$ significantly inhibited the recovery of $CD3^+$, $CD4^+$, $CD8^+$, $CD56^+/CD3^-$ and $CD16^+/CD3^-$ lymphocytes compared with numbers found in patients who had no further post-transplant treatment, but had no effect on the recovery of $CD19^+$ cells. Among patients who did not receive IFN-$\alpha_{2\beta}$, the number of $CD8^+$, $CD56^+/CD3^-$ and $CD16^+CD3^-$ lymphocytes recovered to values similar to normal volunteers with increasing time after intensive therapy, however the number of $CD4^+$ cells remained significantly below levels found in normal volunteers. Although $CD16^+/CD3^-$ and $CD56^+/CD3^-$ cell numbers were reduced in patients receiving IFN-$\alpha_{2\beta}$, natural killer (NK) activity was not effected. The levels of soluble interleukin 2 receptor (sIL-2R) were similar in all patients and IL-2 was not detected in any patient. At the time of writing, of the total of 69 patients, seven have relapsed, of whom three were receiving IFN-$\alpha_{2\beta}$, however there was no correlation between the absolute numbers of any lymphocyte subset with imminent relapse. The data suggest that the recovery of a specific lymphocyte subset(s) in peripheral blood is unlikely to be associated with the maintenance of response after intensive therapy.

**Keywords:** multiple myeloma; interferon $\alpha_{2\beta}$; immune recovery; B cell; NK activity

Multiple myeloma (MM) is a disease of the B-cell lineage and remains incurable. Encouraging results from two clinical studies suggest that maintenance therapy with interferon $\alpha_{2\beta}$ (IFN-$a_{2\beta}$) after intensive therapy with autologous bone marrow rescue (ABMR) (Attal *et al.*, 1992; Cunningham *et al.*, 1993) increases the duration of response and may prolong survival (Cunningham *et al.*, 1993). Since this therapeutic benefit is confined to patients who have had a complete response to treatment, it has been suggested that IFN-$\alpha_{2\beta}$ is only effective in patients who have had a substantial reduction of the tumour burden (Attal *et al.*, 1992). Thus far, there has been no indication that IFN-$\alpha_{2\beta}$ improves the clinical status after intensive therapy, suggesting that the mechanism(s) involved in its therapeutic efficacy is concerned with the maintenance of tumour homeostasis rather than direct cytotoxicity.

The existence of monoclonal gammopathies of undetermined significance (MGUS) and plateau phase MM shows that monoclonal B cells can be kept under homeostatic control *in vivo*. In the case of plateau phase disease, this has been attributed to complex interactions between the malignant clone and the immune system (Joshua, 1988). Several lines of evidence suggest that the evolution of the disease is influenced by the behaviour of immunoregulatory cells. Idiotype-reactive T cells have been found in MM patients with early stage disease (Dianzani *et al.*, 1988; Osterborg *et al.*, 1991) and high levels of interluekin 2 (IL-2) have been correlated with prolonged survival (Cimino *et al.*, 1990). In contrast, high levels of soluble IL 2 receptor (sIL-2R) in peripheral blood correlated with active disease (Vacca *et al.*, 1991) and low $CD4^+$ cell numbers have been associated with both advanced clinical disease and shorter survival (San Miguel *et al.*, 1992).

Massaia *et al.* (1993) have shown that activation of $CD3^+$ lymphocytes from MM patients *in vitro* results in greater production of IFN-$\gamma$ than that produced from normal donor cells. This may have an association with putative tumour control *in vivo* since IFN-$\gamma$, as well as IL-2, induces the differentiation of allergen-specific T cells *in vitro* to TH-1 clones and is induced by IFN-$\alpha$ (Parronchi *et al.*, 1992).

As well as T cell-mediated immunoregulation, there is evidence that natural killer (NK) and lymphocyte-activated killer (LAK) cells may play a role in the regulation of MM. Although the number of precursors of cytotoxic cells may be reduced in patients with MM (Massaia *et al.*, 1988) and LAK activity of peripheral blood T- and non-T lymphocytes is reduced compared with normal donors (Massaia *et al.*, 1989), both cellular and humoral effector mechanisms are retained that can regulate the behaviour of MM cells in model systems (Abbas, 1979; Rohrer and Lynch, 1979). Despite a reduction in NK activity in patients with advanced MM (Osterborg *et al.*, 1990) a signifigant increase has been found in patients with early stage disease (Gonzalez *et al.*, 1992) and in patients following response to treatment or during plateau phase (Osterborg *et al.*, 1990). *In vitro* exposure of peripheral blood mononuclear cells from MM patients to IFN-$\alpha_{2\beta}$ increases NK activity (Uchida *et al.*, 1984) and *in vivo* administration of IFN-$\alpha_{2\beta}$ increased NK activity in patients given the cytokine as sole treatment (Einhorn *et al.*, 1982).

The aim of this study was to determine whether the recovery of one or more lymphocyte subsets associated with immune regulation is enhanced in patients receiving high-dose melphalan (HDM) in combination with ABMR or peripheral blood stem cell rescue (PBSCR) followed by maintenance therapy with IFN-$\alpha_{2\beta}$ and may serve as a measure of the therapeutic effect(s) of the cytokine. Comparisons have been made between patients who are receiving IFN-$\alpha_{2\beta}$ with those who have had no further treatment after intensive therapy and with normal volunteers. In addition, the clinical response of patients to HDM followed by IFN-$\alpha_{2\beta}$ has been monitored to determine whether the cytokine improves the anti-tumour effect of intensive therapy.

### Materials and methods

### *Clinical samples*

Samples (10 ml) of clotted and heparinised peripheral blood were taken at out-patient clinics from patients and normal

Correspondence: BC Millar, Section of Academic Haematology, The McElwain Laboratories, Institute of Cancer Research, 15 Cotswold Road, Belmont, Sutton, Surrey SM2 5NG, UK
Received 30 May 1995; revised 21 August 1995; accepted 22 August 1995

volunteers after informed consent and approval by the local Royal Marsden Hospital Ethics Committee. All patients had received treatment with one of two conditioning regimens, either CY-VAMP [i.v. infusion of vincristine (0.4 mg) and doxorubicin (9 mg $m^{-2}$) over 24 h for 4 days with bolus of methylprednisolone (1.5 g i.v. or orally for 5 days) plus cyclophosphamide (500 mg i.v. bolus on days 1, 8 and 15)] or VERCY-VAMP (vincristine, doxorubicin, methylprednisolone and cyclophosphamide (doses as before) plus verapamil (10 mg i.v. over 24 h for 5 days) followed by HDM either 200 mg $m^{-2}$ with ABMR or PBSCR or 140 mg $m^{-2}$ alone. Patients who had ABMR were given methylprednisolone (1 g $m^{-2}$ daily for 5 days) after HDM. In the total group of 69 patients, two received high-dose busulphan (HDBu) (16 mg $kg^{-1}$ body weight over 4 days) with ABMR, seven were given IFN-$\alpha_{2\beta}$ after CY-VAMP, one received HDM and two received HDM with methylprednisolone without rescue. When their leucocyte count was greater than $3 \times 10^9$ $l^{-1}$ and platelets were greater than $100 \times 10^9$ $l^{-1}$, patients were given IFN-$\alpha_{2\beta}$ (Intron-A, Shering-Plough) at $3 \times 10^6$ U $m^{-2}$ subcutaneously three times weekly until relapse.

The distribution of treatment protocols of patients who had received IFN-$\alpha_{2\beta}$ for more or less than 12 months was similar to that of patients who had no further treatment in each group.

*Clinical status*

A complete remission (CR) was defined as the absence of measurable paraprotein and bone marrow infiltration by myeloma cells of <5%. A partial response (PR) was defined as a paraprotein level reduced by 50% and improvement in all other clinical features sustained for greater than 1 month.

*Flow cytometric analysis*

All blood samples were counted and used for flow cytometric analysis within 2 h of collection. Aliquots of 50 $\mu$l were dispensed into tubes with 10 $\mu$l of ready-conjugated antibody agitated on a mixer and incubated at 4°C for 20 min. Lysis solution (1 ml) (Ortho-Diagnostics, UK) was added to each tube and incubation continued at room temperature for 10 min. The tubes were transferred to an ice bath and analysed immediately. The antibodies used were mouse anti-human CD45-FITC/CD14-RPE (Sigma, UK), CD4-FITC/CD8-RPE (Sigma, UK), CD3-FITC, CD19-RPE, CD16-RPE and CD56-RPE (Becton Dickinson, UK).

Analysis was done using an Ortho Cytoronabsolute (OrthoDiagnostics, UK) with gating for lymphocytes. The machine was calibrated before use with normal donor blood that had been assessed for the different count. Calibration for the lymphocyte gate ensured that >99% of the gated cells were lymphocytes. Data with experimental samples were collected in list mode file and analysed using the software package supplied by the manufacturer. All subsequent calculations were based on the total white cell count assayed by Coulter Counter to provide absolute counts of individual lymphocyte subsets.

*NK activity*

Freshly harvested lymphocytes obtained by Ficoll–Hypaque separation of whole blood (Boyum, 1968) followed by removal of phagocytic cells with carbonyl iron were used as effector cells. The percentage of lymphocytes in the resulting suspensions ranged from 85% to 99%. The K562 cell line was used as target cells for NK activity. Cells were labelled with 200 $\mu$Ci $10^{-6}$ cells of $^{51}$Cr (1 mCi $ml^{-1}$ Amersham International, UK) in a volume of 200 $\mu$l. Samples were tested at four effector-target (E/T) cell ratios (60:1, 30:1, 15:1 and 7.5:1) using 5000 K562 cells per well in a total volume of 200 $\mu$l in 96-well microtitre plates. Samples were assayed in triplicate. The cell mixtures were incubated for 18 h at 37°C. The radioactivity in the supernatant was analysed in a gamma counter (Hydragamma 16, Innotron, UK). Spontaneous release was determined by incubation of $^{51}$Cr-labelled cells with medium alone. Maximum release was measured by incubating the cells with lysis buffer (20 mM Hepes pH 6.9, 5 mM potassium chloride, 5 mM magnesium acetate, 1 mM DTT and 0.5% Nonidet 140). The percentage of $^{51}$Cr released was calculated from the formula;

$$\% \quad \text{release} = \frac{(\text{experimental } ^{51}\text{Cr release} - \text{spontaneous } ^{51}\text{Cr release})}{(\text{total } ^{51}\text{Cr release} - \text{spontaneous } ^{51}\text{Cr release}} \times 100$$

Absolute numbers of $CD16^+/CD3^-$ and $CD56^+/CD3^-$ lymphocytes were calculated after depletion of phagocytic cells using flow cytometry as above. The cells were labelled with CD45-FITC/CD14-RPE, CD3-FITC/CD16-RPE and CD3-FITC/CD56-RPE.

*Detection of IL-2 and sIL-2R*

Samples of clotted blood were separated and the serum stored at −20° until use. IL-2 and IL-2R were measured using enzyme-linked assays (Quantikine, R & D Systems, USA, and Boehringer Mannheim Biochemica, UK, respectively). Assays were performed in accordance with the manufacturer's instructions.

*Statistics*

Data were analysed using a non parametric Mann-Whitney *U*-test for significance. *P*-values from two-tailed Mann-Whitney *U*-tests are given in the text.

### Results

*Lymphocyte analysis*

The clinical status of MM patients used in this study at the time of examining the lymphocyte subsets is shown in Table I. Although total white cell counts (WBCs) remained significantly lower in patients who received IFN-$\alpha_{2\beta}$ for prolonged periods after intensive therapy than in patients who had no further treatment ($P<0.02$) or normal volunteers ($P<0.0002$), this was not a reflection of a general inhibition of recovery among each of the lymphocyte subsets (Table II). Despite a significant reduction in B-cell numbers in all patients after HDM, IFN-$\alpha_{2\beta}$ had no effect on their recovery compared with that seen among patients who had no further treatment, and numbers returned to levels similar to those in normal volunteers 12 months after HDM. In contrast, IFN-$\alpha_{2\beta}$ inhibited the recovery of $CD3^+$, $CD4^+/CD3^+$, $CD8^+/CD3^+$, $CD16^+/CD3^-$ and $CD56^+/CD3^-$ lymphocytes. Since HDM also inhibited the recovery of $CD4^+/CD3^+$ T cells, the poor recovery in this subset was augmented by IFN-$\alpha_{2\beta}$. The data are summarised in Table II.

*NK activity*

NK activity correlated with absolute numbers of $CD16^+/CD3^-$ lymphocytes ($r=0.616$) (Figure 1) but not with $CD56^+/CD3^-$ cells (data not shown). Although absolute numbers of both $CD16^+/CD3^-$ and $CD56^+/CD3^-$ lymphocytes remained low in patients receiving long-term treatment with IFN-$\alpha_{2\beta}$ (Table II), there was no significant difference between NK activity in patients or normal volunteers. The mean lytic activity was 60%±6.2% in normal volunteers, 57.8%±4.2% in patients who had no further treatment and 51.75%±6.0% in patients on IFN-$\alpha_{2\beta}$ (Figure 2).

*sIL-2R and IL-2*

Analysis of sIL-2R in serum from the patients who were taking IFN-$\alpha_{2\beta}$ compared with those who were not showed that there was no significant difference between the levels compared with that reported for normal volunteers. The

**Table I** Clinical status of multiple myeloma patients treated with HDM/ABMR or PBSCR with or without maintenance therapy with interferon $\alpha_{2b}$

| | *Median time after HDM in months (range)* | *Median time on interferon $\alpha_{2\beta}$ in months (range)* | *Clinical status[a] at time of testing* | | | | *Change in clinical status to present (July 1995)* | *Time of status change after HDM* |
|---|---|---|---|---|---|---|---|---|
| | | | *CR* | *PR* | *NC* | *PD* | | |
| No interferon $\alpha_{2\beta}$ group < 12 months ($n$ = 10) | 3.5 (1–10) | NA† | 1 | 5 | 3 | 1 | PR→CR | – |
| | | | | | | | CR→PD | 6 months |
| | | | | | | | PR→PD | 11 months |
| No interferon $\alpha_{2\beta}$ group > 12 months ($n$ = 10) | 47 (20–103) | NA | 6 | 2 | 1 | 1 | CR→PD | 61 months |
| | | | | | | | PR→PD | 107 months |
| Interferon $\alpha_{2\beta}$ group < 12 months ($n$ = 28) | 5 (2–13) | 3 (0.5–12) | 11 | 11 | 6 | 0 | PR→CR | – |
| | | | | | | | PR→CR | – |
| | | | | | | | NC→CR | – |
| | | | | | | | CR→PD | 6 months |
| | | | | | | | CR→PD | 9 months |
| | | | | | | | PR→PD | 12 months |
| Interferon $\alpha_{2\beta}$ group > 12 months ($n$ = 21) | 46 (21–102) | 27 (13–70) | 11 | 6 | 3 | 1 | No change in status of any patient | – |

[a]CR, complete remission; PR, partial response; NC, no change after disease progression; PD, progressive disease (samples taken on day this was identified). NA, not applicable.

**Table II** Comparison of surface epitopes in peripheral blood from normal donors with multiple myeloma patients treated with HDM/ABMR or PBSCR with or without maintenance therapy with interferon-$\alpha_{2\beta}$ at time intervals of 1–12 months and more than 12 months therapy

| | *Normal donors* (n = *10*) | *MM patients without IFN therapy* *1–12 months after HDM* (n = *10*) | *MM patients without IFN therapy* *> 12 months after HDM* (n = *10*) | *MM patients given IFN therapy* *1–12 months on IFN* (n = *28*) | *MM patients given IFN therapy* *> 12 months on IFN* (n = *21*) |
|---|---|---|---|---|---|
| WBC | 7.43 ± 0.60 | 4.47 ± 0.50 | 6.60 ± 0.87 | 3.98 ± 0.34 | 4.51 ± 0.32[a] |
| $CD19^+$ | 0.221 ± 0.043 | 0.056 ± 0.019[b] | 0.396 ± 0.092 | 0.100 ± 0.016[b] | 0.275 ± 0.038 |
| $CD3^+$ | 1.506 ± 0.181 | 0.959 ± 0.196 | 1.252 ± 0.232 | 0.572 ± 0.037[c] | 0.612 ± 0.062[c,d] |
| $CD16^+/CED3^-$ | 0.179 ± 0.034 | 0.147 ± 0.049 | 0.193 ± 0.039 | 0.058 ± 0.010 | 0.079 ± 0.010[e] |
| $CD56^+/CD3^-$ | 0.329 ± 0.035 | 0.243 ± 0.060 | 0.342 ± 0.068 | 0.115 ± 0.015 | 0.204 ± 0.022[f] |
| $CD8^+$ | 0.610 ± 0.094 | 0.741 ± 0.158 | 0.724 ± 0.156 | 0.402 ± 0.032 | 0.305 ± 0.036[g] |
| $CD4^+$ | 0.894 ± 0.128 | 0.218 ± 0.044[h] | 0.528 ± 0.093[i] | 0.175 ± 0.016[h] | 0.306 ± 0.032[j] |

Results are given as absolute counts $\times 10^6$ ml$^{-1}$ (mean ± s.e.) [a]$P < 0.0002$ *vs* normal donors; $P < 0.02$ *vs* patients who had no further treatment (NFT). [b]$P < 0.005$ *vs* normal donors. [c]$P < 0.00001$ *vs* normal donors. [d]$P < 0.01$ *vs* NFT. [e]$P < 0.02$ *vs* normal donors. [f]$P < 0.01$ *vs* normal donors. [g]$P < 0.005$ *vs* normal donors. $P < 0.01$ *vs* NFT. [h]$P < 0.00005$ *vs* normal donors. [i]$P < 0.05$ *vs* normal donors. [j]$P < 0.02$ *vs* NFT.

**Figure 1** Correlation of NK activity with absolute cell numbers of $CD16^+/CD3^-$ cells. ●, Normal volunteers; △, myeloma patients on interferon $\alpha_{2\beta}$; ▲, myeloma patients not on interferon $\alpha_{2\beta}$. NK activity is expressed as per cent lysis of 5000 $^{51}$Cr-labelled K562 cells.

**Figure 2** Comparison of NK activity in lymphocytes from normal volunteers (●), myeloma patients on maintenance therapy with interferon $\alpha_{2\beta}$ (△) and myeloma patients not having maintenance therapy (▲). NK activity is expressed as per cent lysis of 5000 $^{51}$Cr-labelled K562 cells; bar indicates mean value.

mean level was 116.7 ± 10.1 pmol l$^{-1}$ in patients receiving the cytokine compared with 109.4 ± 15.6 pmol l$^{-1}$ in patients who had no further treatment. IL-2 was not detected in serum from any patient.

### *Clinical response*

At the time of writing, 7/69 patients have relapsed within 6 months after examining their lymphocyte subsets, three of whom were receiving IFN-$\alpha_{2\beta}$. Although $CD19^+$ cell numbers were lower in patients who relapsed subsequently, this was not statistically significant. There was no correlation between absolute numbers of other lymphocyte subsets among patients who relapsed and those in patients who remain in remission, irrespective of treatment with IFN-$\alpha_{2\beta}$.

### Discussion

Although IFN-$\alpha_{2\beta}$ has been used as maintenance therapy after HDM for more than 6 years at the Royal Marsden Hospital, the mechanism by which it regulates the proliferation of MM remains elusive. Examination of peripheral blood samples after short (median time, 3 months) and long (>12 months) exposure to IFN-$\alpha_{2\beta}$ failed to identify the enhanced recovery of a specific lymphocyte subset(s) which might be a prognostic indicator for the anti-proliferative effect of IFN-$\alpha_{2\beta}$.

Several authors have reported that continuous treatment with IFN-$\alpha_{2\beta}$ suppresses the peripheral blood WBC but that recovery is rapid following cessation of treatment (Klingemann *et al.*, 1991). Reduced numbers of most lymphocyte subsets contributed significantly to the delayed recovery of the WBC in patients receiving IFN-$\alpha_{2\beta}$ in addition to a long-time inhibitory effect on the recovery of $CD4^+$ cell caused by therapy (Bergmann *et al.*, 1984). However, despite the reduction in B cell numbers in all patients during the first 12 months after intensive therapy, their subsequent recovery was not inhibited by IFN-$\alpha_{2\beta}$ and cell numbers were similar to those in normal volunteers at longer time intervals.

Previous studies have considered the role of immunoregulatory cells as prognostic indicators in untreated patients with MM (Massaia *et al.*, 1988, 1989) and the effects of IFN-$\alpha_{2\beta}$ on these cells *in vitro* (Uchida *et al.*, 1984) or as a single agent *in vivo* (Einhorn *et al.*, 1982). The association of low numbers of $CD4^+$ lymphocytes with poor prognosis (Bergmann *et al.*, 1984) is clearly inappropriate as a criterion of imminent relapse after intensive therapy, since their recovery is known to be inhibited by chemotherapy (Bergmann *et al.*, 1984). In our patients, the delayed recovery of $CD4^+$ lymphocytes was increased by IFN-$\alpha_{2\beta}$ whereas the number of $CD8^+$ cells was similar to those of normal volunteers in patients who had no further treatment within 12 months after intensive therapy. These data suggest that despite the differential sensitivity of $CD4^+$ and $CD8^+$ lymphocytes to chemotherapy they are equally sensitive to the anti-proliferative effects of IFN-$\alpha_{2\beta}$.

Although increased numbers of activated NK cells have been found in the peripheral blood of untreated patients with early stage disease (Osterborg *et al.*, 1990; Gonzalez *et al.*, 1992), there was no indication that the magnitude of the IFN-$\alpha_{2\beta}$-induced enhancement of NK activity correlated with clinical response to IFN-$\alpha_{2\beta}$ (Einhorn *et al.*, 1982). While the latter study questions the role of NK cells *in vivo* in response to IFN-$\alpha_{2\beta}$ it is unlikely to be comparable with mechanisms that are operable after intensive therapy. In a study in which IFN-$\alpha_{2\beta}$ was given to patients early after allogeneic transplantation for advanced haematological diseases, NK function was low before IFN and was not improved with the cytokine (Klingemann *et al.*, 1991), however the report took no account of absolute numbers of NK cells. Although recovery of the two major NK subsets, $CD16^+/CD3^-$ and $CD56^+/CD3^-$ cells, was delayed in our patients receiving IFN-$\alpha_{2\beta}$ compared with patients who had no further treatment, there was no difference in NK activity measured by the lysis of K562 cells between patients and normal donors. The data suggest that if NK cells have a role in mediating tumour homeostatis *in vivo* after intensive therapy, the efficacy of this mechanism may be lower in patients receiving IFN-$\alpha_{2\beta}$ because of fewer NK cells.

Given that MM is a disease of the B-cell lineage, the recovery of B-cells in patients receiving IFN-$\alpha_{2\beta}$ raises questions concerning both the putative stem cell in MM as well as the mechanism by which tumour homeostasis is achieved. Montes Borinaga (1993) showed that MM cells can be cultured *in vitro* from patients receiving IFN-$\alpha_{2\beta}$ as maintenance therapy during remission after HDM/ABMR suggesting that the effect of the cytokine *in vivo* is likely to be cytostatic rather than cytocydal. Although the stem cell in MM has not been identified several authors agree that it is likely to arise after class-switching immediately before or at the level of the memory B-cell (Ralph *et al.*, 1993; Sahota *et al.*, 1994). The failure of neutralising antibody to IFN-$\alpha_{2\beta}$ to persist in patients receiving the cytokine as maintenance therapy led to the suggestion that memory B-cells may not be involved (Millar and Bell, 1995). An alternative conclusion could be that the proliferation and maturation of memory cells may be inhibited in patients receiving the cytokine. Despite our failure to identify a particular lymphocyte subset in the peripheral blood that may effect tumour homeostasis, we cannot exclude the possibility that changes may have occurred in the milieu of the bone marrow or lymph nodes that might account for inhibition of tumour cell proliferation. Although sequestration of cytotoxic T cells and/or NK cells to those sites may be responsible for localised control, the relapse of three patients while receiving IFN-$\alpha_{2\beta}$ within 6 months of HDM with PBSCR suggets that the efficacy of such a control mechanism would require a fine balance between the residual tumour burden and the anti-tumour effect of the cytokine.

The lack of correlation between absolute numbers of lymphocyte subsets with relapse may be indicative of an alternative mechanism dependent on changes in the cytokine profile at specific sites within the bone marrow or lymph nodes. There is increasing evidence that the cytokine profile of the natural immune response probably determines the phenotype of the specific immune response (Romagini, 1992). For example, Th1 (which produce IL-2 and IFN-$\gamma$) and Th2 (which produce IL-4 and IL-5) lymphocytes are associated with markedly different functions whereas Th0 cells show an unrestricted lymphokine pattern (Firestein *et al.*, 1989). Although IFN-$\alpha_{2\beta}$ promotes the differentiation of allergen-specific T cells into Th1 instead of Th2 clones (Parronchi *et al.*, 1992), IL-2 was not detected in the serum from any patient, neither were the levels of sIL-2R different from those in patients who did not receive the cytokine. However, the net effect of INF-$\alpha_{2\beta}$ *in vivo* may result in the localised release of INF-$\gamma$ and IL-2 by different T cell subsets depending on their proximity to cells that can respond and may be insufficient to be detected in serum.

In conclusion, there was no enhanced recovery of a particular lymphocyte subset(s) in peripheral blood samples that may account for the anti-tumour effect of IFN-$\alpha_{2\beta}$ as maintenance therapy. It seems likely that the anti-proliferative effect of the cytokine is determined by events that occur at localised sites within the bone marrow or lymph nodes and that such events are dependent critically on the residual tumour mass.

**Acknowledgements**
We thank the Cancer Research Campaign for funding this work and the staff and patients at the Royal Marsden NH Trust, Sutton, Surrey, for clinical samples.

### References

ABBAS AK. (1979). T-lymphocyte-mediated suppression of myeloma function *in vitro*. I. Suppression by allogeneically activated T-lymphocytes. *J. Immunol.*, **123**, 2011–2018.

ATTAL M, HUGUET F, SCHLAIFER D, PAYEN C, LAROCHE M, FOURNIE B, MAZIERES B, PRIS J AND LAURENT G. (1992). Intensive combined therapy for previously untreated aggressive myeloma. *Blood*, **79**, 1130–1136.

BERGMANN L, MITRON PS, WEBER KC, KELKER W. (1984). Imbalances of T-cell subsets in monoclonal gammopathies. *Cancer Immunol. Immunother.*, **17**, 112–116.

BOYUM A. (1968). Separation of leucocytes from blood and bone marrow. *Scand. J. Clin. Lab. Invest.*, **21**, 1–6.

CIMINO G, AVVISATA G, AMADORI S, CAVA MC, GIANARELLI D, DINUCCI GD, MAGLIOCCI V, PETRUCCI MT, POTI G, SGADARI C AND MANDELLI F. (1990). High serum IL-2 levels are predictive of prolonged survival in multiple myeloma. *Br. J. Haematol.*, **75,** 373-377.
CUNNINGHAM D, POWLES R, MALPAS JS, MILAN S, MELDRUM M, VINER C, MONTES A, HICKISH T, NICOLSON M, JOHNSON P, MANSI J, TRELEAVEN J, RAYMOND J AND GORE ME. (1993). A randomised trial of maintenance therapy with Intron A following high dose melphalan and ABMT in myeloma. *Br. J. Cancer*, **67** (suppl.XX), 30.
DIANZANI U, PIERI A, BOCCADORO M, PALUMBO A, PIOPPO P, BIANCHI A, CAMPONI A, BATTAGLIO S AND MASSAIA M. (1988). Activated idiotype-reactive cells in suppressor/cytotoxic subpopulations of monoclonal gammopathies; correlation with diagnosis and disease status. *Blood* **72,** 1064-1068.
EINHORN S, AHRE A, BLOMGREN H, JOHANSSON B AND MELLSTEDT H. (1982). Interferon and natural killer activity in multiple myeloma. Lack of correlation between interferon-induced enhancement of natural killer activity and clinical response to human interferon-$\alpha$. *Int. J. Cancer*, **30,** 167-172.
FIRESTEIN GS, ROEDER WD, LAXER JA, TAMSEND KS, WEAVER CT, HOM JT, LINTON J, TORBETH BE AND CASEBROOK AC. (1989). A new murine $CD4^+$ T-cell subset with unrestricted cytokine profile. *J. Immunol.*, **143,** 518-525.
GONZALEZ M, SAN MIGUEL JF, GASCON A, MORO MJ, HERNANDEZ JM, ORTEGA F, TIMENEZ R, GUERRAS L, ROMERO M, CASANOVA F, SANZ MA, PORTERO JA AND ORFAO A. (1992). Increased expression of natural killer associated and activation antigens in multiple myeloma. *Am. J. Hematol.*, **37,** 84-89.
JOSHUA DE. (1988). Biology of multiple myeloma. Host tumour interactions and immunoregulation of disease activity. *Hematol. Oncol.*, **6,** 83-88.
KLINGEMANN HG, GRIGG AP, BOYD KW, BARNETT MJ, EAVES AC, REECE DE, SHEPHERD JD AND PHILLIPS GL. (1991). Treatment with recombinant interferon ($\alpha_{2\beta}$) early after bone marrow transplantation in patients at high risk for relapse. *Blood*, **78,** 3306-3311.
MASSAIA M, DIAZANI U, BIANCHI A, CAMPONI A, BOCCADORO M AND PILERI A. (1988). Defective generation of alloreactive cytotoxic T lymphocytes (CTL) in human monoclonal gammopathies. *Clin. Exp. Immunol.*, **73,** 214-218.
MASSAIA M, BIANCHI A, DIAZANI U. (1989). Defective interleukin 2 induction of lymphokine-activated killer (LAK) activity in peripheral blood T lymphocytes of patients with monoclonal gammopathies. *Clin. Exp. Immunol.*, **79,** 100-104.
MASSAIA M, ATTISANO C, PEOLA S, MONTACCHINI L, OMEDE P, CORRADINI F, FERRERO D, BOCCADORO M, BIANCHI A AND PILERI A. (1993). Rapid generation of antiplasma cell activity in the bone marrow of myeloma patients by CD3 activated T-cells. *Blood*, **82,** 1787-1797.
MILLAR BC AND BELL JBG. (1995). 2′-5′ Oligoadenylate synthetase levels in patients with multiple myeloma receiving maintenance therapy with interferon-$\alpha_{2\beta}$. *Br. J. Cancer*, **72,** 1525-1530.
MONTES BORINAGA A. (1993). Biological studies with growth factors in multiple myeloma; the effect of IL-6 and alpha interferon on the proliferation of multiple myeloma *in vitro* and the clinical efficacy of alpha interferon *in vivo*. MD Thesis, Universidad del Pais Vasco, Leioa, Mayo, Spain.
OSTERBORG A, NILSSON B, BJORKHOLM M, HOLM G AND MELLSTEDT H. (1990). Natural killer cell activity in monoclonal gammopathies: relation to disease activity. *Eur. J. Haematol.*, **45,** 153-157.
OSTERBORG A, MASUCCI M, BERGENBRANT S, HOLM G, LEFVERT AK AND MELLSTEDT H. (1991). Generation of T-cell clones binding $F(ab')_2$ fragments of the idiotypic immunoglobulin in patients with monoclonal gammopathy. *Cancer Immunol. Immunother.*, **34,** 157-162.
PARRONCHI P, DECARLI M, MANETTI R, SIMONELLI C, SAMPOGNAROS S, PICCINI MP, MACCHIA E, MAGGI E, DEL PRETE G AND ROMAGNANI S. (1992). IL-4 and IFN ($\alpha$ and $\gamma$) exert opposite regulatory effects on the development of cytolytic potential by TH-1 and TH-2 human T-cell clones. *J. Immunol.*, **149,** 2977-2983.
RALPH QM, BRISCO MJ, JOSHUA DE, BROWN R, GIBSON J AND MORLEY AA. (1993). Advancement of multiple myeloma from diagnosis through plateau phase to progression does not involve a new B-cell clone: Evidence from the Ig heavy chain gene. *Blood*, **82,** 202-206.
ROHRER JW AND LYNCH RG. (1979). Immunoregulation of localised and disseminated multiple myeloma: antigen-specific regulation of MOPC 315 stem cell proliferation and secretory cell differentiation. *J. Immunol.*, **123,** 1083-1087.
ROMAGNINI S. (1992). Induction of Th1 and Th2 responses: a key role for the 'natural' immune response? *Immunol. Today.*, **13,** 379-381.
SAHOTA S, HAMBLIN T, OSCIER DG AND STEVENSON FK. (1994). Assessment of the role of clonogenic B lymphocytes in the pathogenesis of multiple myeloma. *Leukemia*, **8,** 1285-1289.
SAN MIGUEL JF, GONZALEZ M, GASCON A, MORO MJ, HERNANDEZ JM, ORTEGA F, JIMENEZ R, GUERRAS L, ROMERO M AND CASANOVA F. (1992). Lymphoid subsets and prognosis factors in multiple myeloma. Cooperative group for the study of monoclonal gammopathies. *Br. J. Haematol.*, **80,** 305-309.
UCHIDA AA, YAGITA M, SUGIYAMA H, HOSHINO T AND MOORE M. (1984). Strong natural killer (NK) cell activity in bone marrow of myeloma patients: Accelerated maturation of bone marrow NK cells and their interaction with other bone marrow cells. *Int. J. Cancer*, **34,** 375-381.
VACCA A, DI STEFANO R, FRASSANITO A, IODICE G AND DAMMACCO F. (1991). A disturbance of the IL-2/IL-2 receptor system parallels the activity of multiple myeloma. *Clin. Exp. Immunol.*, **84,** 429-434.


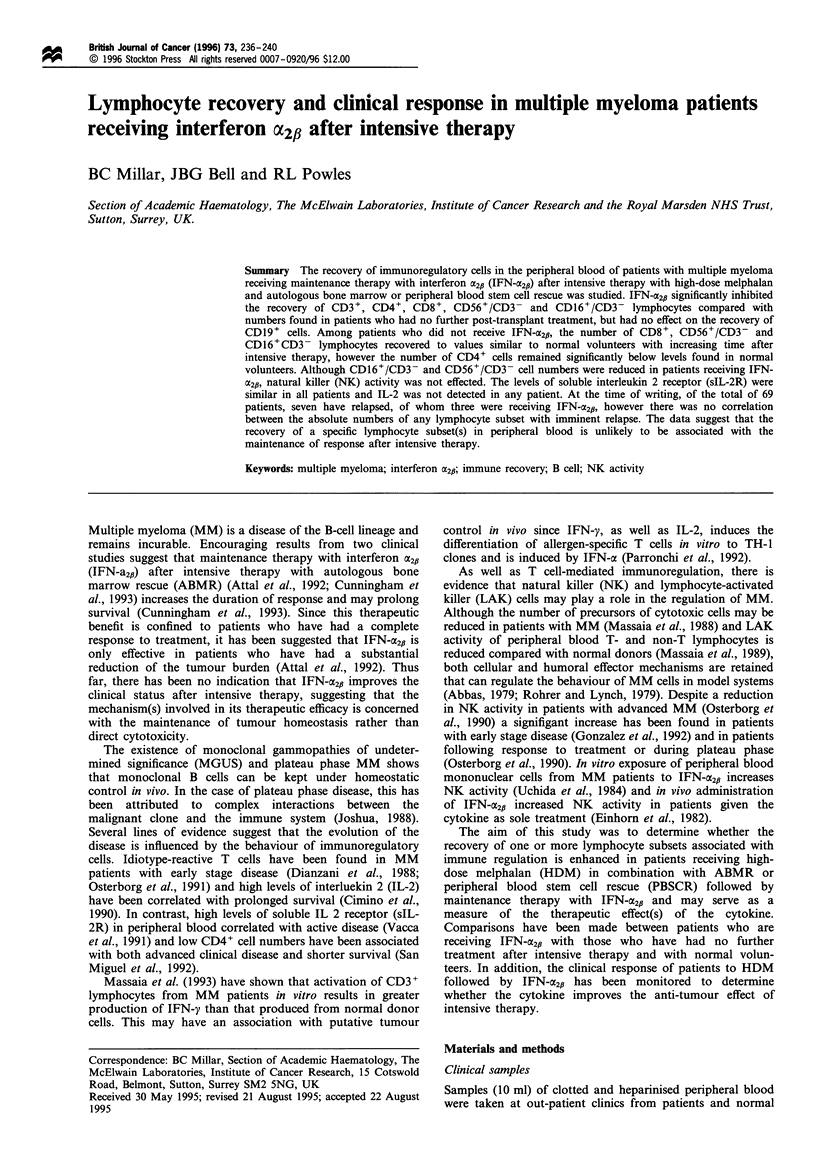

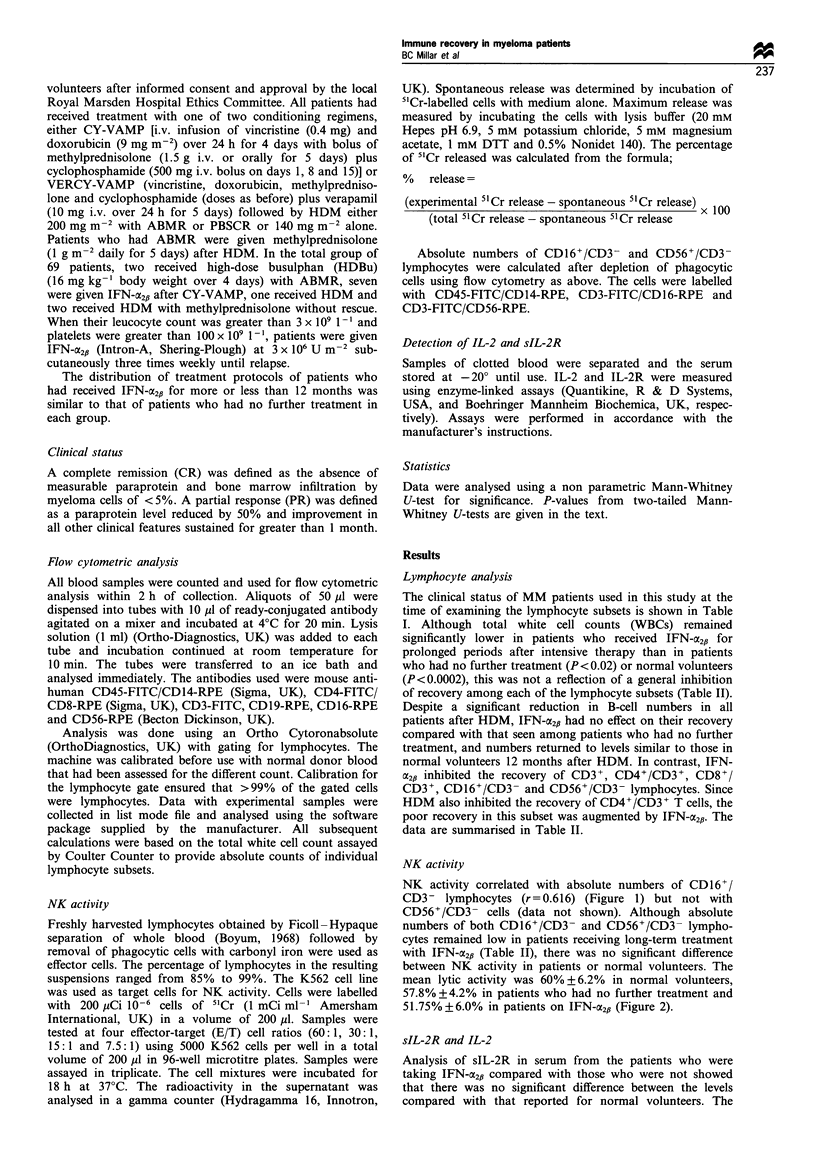

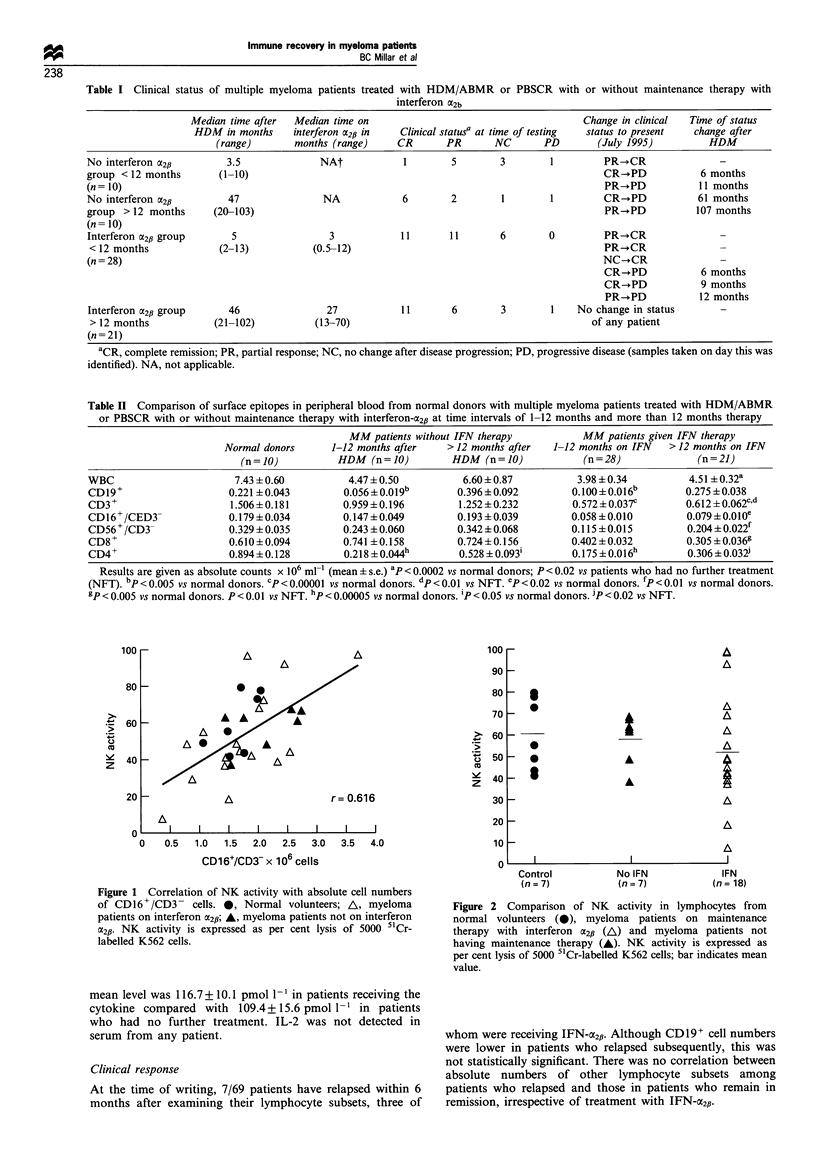

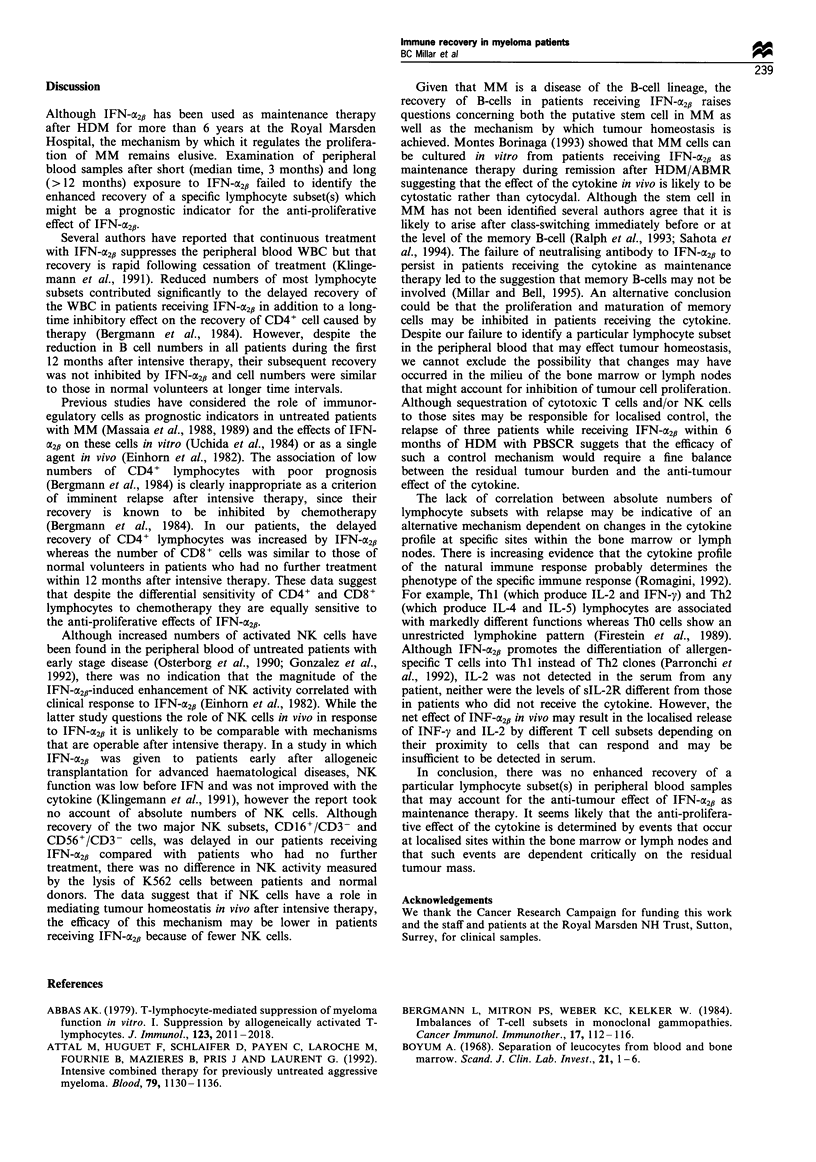

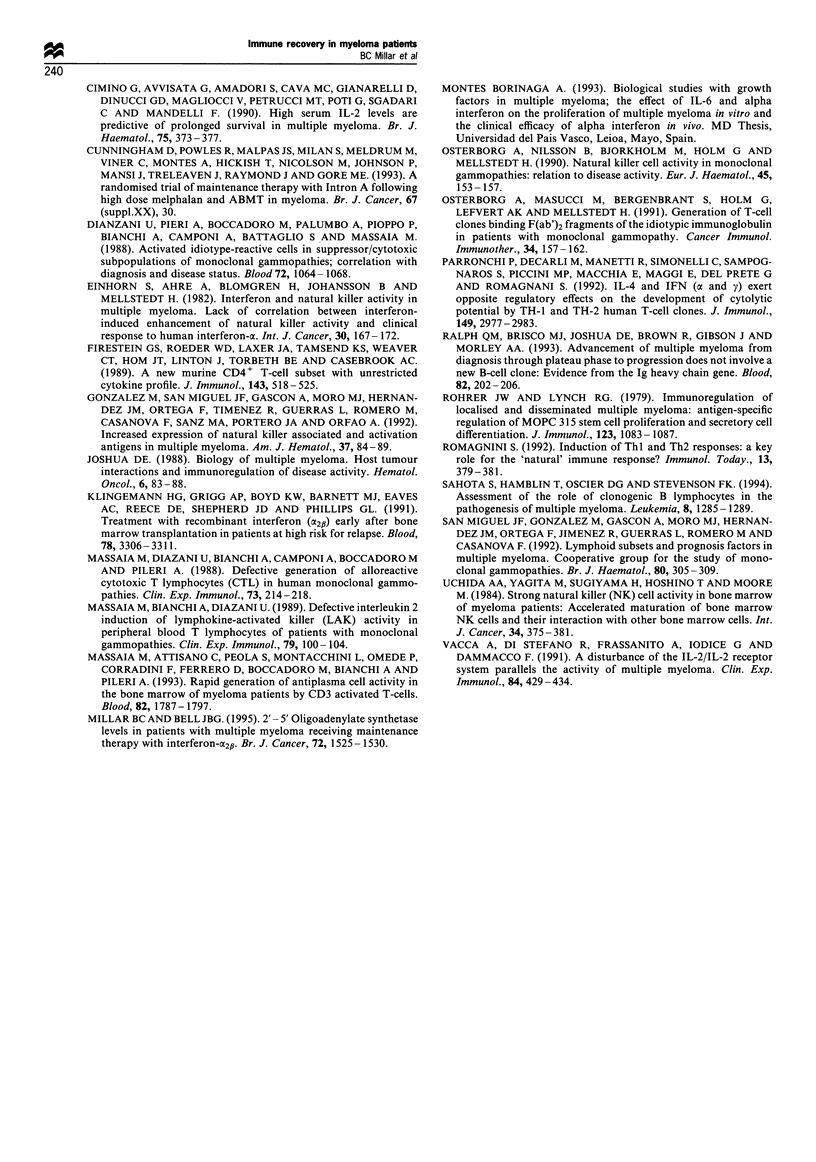


## References

[OCR_00618] Abbas A. K. (1979). T lymphocyte-mediated suppression of myeloma function in vitro. I. Suppression by allogeneically activated T lymphocytes.. J Immunol.

[OCR_00625] Attal M., Huguet F., Schlaifer D., Payen C., Laroche M., Fournie B., Mazieres B., Pris J., Laurent G. (1992). Intensive combined therapy for previously untreated aggressive myeloma.. Blood.

[OCR_00629] Bergmann L., Mitrou P. S., Weber K. C., Kelker W. (1984). Imbalances of T-cell subsets in monoclonal gammopathies.. Cancer Immunol Immunother.

[OCR_00646] Cimino G., Avvisati G., Amadori S., Cava M. C., Giannarelli D., Di Nucci G. D., Magliocca V., Petrucci M. T., Poti G., Sgadari C. (1990). High serum IL-2 levels are predictive of prolonged survival in multiple myeloma.. Br J Haematol.

[OCR_00658] Dianzani U., Pileri A., Boccadoro M., Palumbo A., Pioppo P., Bianchi A., Camponi A., Fossati G., Battaglio S., Massaia M. (1988). Activated idiotype-reactive cells in suppressor/cytotoxic subpopulations of monoclonal gammopathies: correlation with diagnosis and disease status.. Blood.

[OCR_00668] Einhorn S., Ahre A., Blomgren H., Johansson B., Mellstedt H., Strander H. (1982). Interferon and natural killer activity in multiple myeloma. Lack of correlation between interferon-induced enhancement of natural killer activity and clinical response to human interferon-alpha.. Int J Cancer.

[OCR_00674] Firestein G. S., Roeder W. D., Laxer J. A., Townsend K. S., Weaver C. T., Hom J. T., Linton J., Torbett B. E., Glasebrook A. L. (1989). A new murine CD4+ T cell subset with an unrestricted cytokine profile.. J Immunol.

[OCR_00681] Gonzalez M., San Miguel J. F., Gascon A., Moro M. J., Hernandez J. M., Ortega F., Jimenez R., Guerras L., Romero M., Casanova F. (1992). Increased expression of natural-killer-associated and activation antigens in multiple myeloma.. Am J Hematol.

[OCR_00687] Joshua D. E. (1988). Biology of multiple myeloma--host-tumour interactions and immune regulation of disease activity.. Hematol Oncol.

[OCR_00693] Klingemann H. G., Grigg A. P., Wilkie-Boyd K., Barnett M. J., Eaves A. C., Reece D. E., Shepherd J. D., Phillips G. L. (1991). Treatment with recombinant interferon (alpha-2b) early after bone marrow transplantation in patients at high risk for relapse [corrected].. Blood.

[OCR_00712] Massaia M., Attisano C., Peola S., Montacchini L., Omedé P., Corradini P., Ferrero D., Boccadoro M., Bianchi A., Pileri A. (1993). Rapid generation of antiplasma cell activity in the bone marrow of myeloma patients by CD3-activated T cells.. Blood.

[OCR_00705] Massaia M., Bianchi A., Dianzani U., Camponi A., Attisano C., Boccadoro M., Pileri A. (1990). Defective interleukin-2 induction of lymphokine-activated killer (LAK) activity in peripheral blood T lymphocytes of patients with monoclonal gammopathies.. Clin Exp Immunol.

[OCR_00700] Massaia M., Dianzani U., Bianchi A., Camponi A., Boccadoro M., Pileri A. (1988). Defective generation of alloreactive cytotoxic T lymphocytes (CTL) in human monoclonal gammopathies.. Clin Exp Immunol.

[OCR_00718] Millar B. C., Bell J. B. (1995). 2',5'-Oligoadenylate synthetase levels in patients with multiple myeloma receiving maintenance therapy with interferon alpha 2b do not correlate with clinical response.. Br J Cancer.

[OCR_00737] Osterborg A., Masucci M., Bergenbrant S., Holm G., Lefvert A. K., Mellstedt H. (1991). Generation of T cell clones binding F(ab')2 fragments of the idiotypic immunoglobulin in patients with monoclonal gammopathy.. Cancer Immunol Immunother.

[OCR_00731] Osterborg A., Nilsson B., Björkholm M., Holm G., Mellstedt H. (1990). Natural killer cell activity in monoclonal gammopathies: relation to disease activity.. Eur J Haematol.

[OCR_00745] Parronchi P., De Carli M., Manetti R., Simonelli C., Sampognaro S., Piccinni M. P., Macchia D., Maggi E., Del Prete G., Romagnani S. (1992). IL-4 and IFN (alpha and gamma) exert opposite regulatory effects on the development of cytolytic potential by Th1 or Th2 human T cell clones.. J Immunol.

[OCR_00751] Ralph Q. M., Brisco M. J., Joshua D. E., Brown R., Gibson J., Morley A. A. (1993). Advancement of multiple myeloma from diagnosis through plateau phase to progression does not involve a new B-cell clone: evidence from the Ig heavy chain gene.. Blood.

[OCR_00756] Rohrer J. W., Lynch R. G. (1979). Immunoregulation of localized and disseminated murine myeloma: antigen-specific regulation of MOPC-315 stem cell proliferation and secretory cell differentiation.. J Immunol.

[OCR_00769] Sahota S., Hamblin T., Oscier D. G., Stevenson F. K. (1994). Assessment of the role of clonogenic B lymphocytes in the pathogenesis of multiple myeloma.. Leukemia.

[OCR_00772] San Miguel J. F., González M., Gascón A., Moro M. J., Hernández J. M., Ortega F., Jiménez R., Guerras L., Romero M., Casanova F. (1992). Lymphoid subsets and prognostic factors in multiple myeloma. Cooperative Group for the Study of Monoclonal Gammopathies.. Br J Haematol.

[OCR_00779] Uchida A., Yagita M., Sugiyama H., Hoshino T., Moore M. (1984). Strong natural killer (NK) cell activity in bone marrow of myeloma patients: accelerated maturation of bone marrow NK cells and their interaction with other bone marrow cells.. Int J Cancer.

[OCR_00786] Vacca A., Di Stefano R., Frassanito A., Iodice G., Dammacco F. (1991). A disturbance of the IL-2/IL-2 receptor system parallels the activity of multiple myeloma.. Clin Exp Immunol.

